# Shared challenges in psychiatric research in India and Sri Lanka

**DOI:** 10.4103/0019-5545.69217

**Published:** 2010-01

**Authors:** Harischandra Gambheera, Shehan Williams

**Affiliations:** National Institute of Mental Health, Colombo, University of Kelaniya, Sri Lanka; 1 Department of Psychiatry, Faculty of Medicine, University of Kelaniya, Sri Lanka

**Keywords:** India and Sri Lanka, psychiatric research, evidence based medicine

## Abstract

The need for good research in psychiatry has never been more important than in this era of ‘Evidence-based medicine’ (EBM).[1] The countries in south Asia have to rise to the challenge and abandon the emphasis placed on ‘Experiencebased medicine’, as was popular in the traditional systems of medicine – the art was handed down from father to son or guru to shishya (student).

Evidence-based medicine does not abandon clinician experience, skills, and judgment, but rather complements it with the best available evidence and patient choice.[2] This article explores the challenges in obtaining the best available evidence in the south Asian context.

## INTRODUCTION

### The need for quality local research

Good quality research is essential to generate the evidence that can be applied to practice. Finding this evidence is the challenge in the local context. Most studies are conducted in high-income countries, and the findings per se may not be directly relevant. The populations in which the research has been carried out may have different ethnographic, demographic, and sociocultural variables, vastly different from our own. This is pertinent particularly in pharmacological trials, where the pharmacokinetics of a drug can be different from the Caucasian populations where drug trials are conducted.

In terms of practicing EBM[1,2], the determinants of patient choice are also different in a ‘collective culture’ such as ours, where individual choice is not valued. The choice is made in consultation with friends and family, who offer differing views. Patient choice is also determined by the value placed on alternatives to allopathic medicine. This includes faith in religious gurus to astrological predictions or preference for ayurvedic and native treatment methods.

The credible clinician is usually expected to make an informed recommendation, somewhat paternalistically, on the preferred treatment modality. The clinician, however, is often at a loss to make this informed recommendation for want of evidence in the local context.

The research output from low- and middle-income countries, including India and its neighbors, in international journals, remains low.[3,4] How does one then increase relevant research output in the Indian subcontinent that will address the needs of our patients?

### Time constraints

The patients per psychiatrist ratio remains high with 0.25 psychiatrists per 100,000 population.[5] The clinical load of individual psychiatrists is so heavy that most psychiatrists in the subcontinent work long hours, often exceeding 12 hours each day. The pressure to continue clinical work out of hours continues, as they are underpaid within the government health service and even in private clinics charge a low fee as patients cannot afford to pay for their services. In such a situation few psychiatrists have the time or energy to pursue research.

### Training

Investing in training in research methodology is imperative. It is noteworthy that the postgraduate MD programs in India, Pakistan, Bangladesh, and Sri Lanka have a research component. Even as one commends this step, it is important that a healthy research culture is encouraged and research is not conducted by hook or by crook, to meet the eligibility criteria for professional advancement.

One can no longer hide behind the excuse of lack of access to journals and reference material, in defense of poor quality research output. With the expansion of internet access, an increasing number of resources are being made available to researchers in the region. Sadly, few journals from the low-and middle-income countries are indexed, thus denying wider access to research published in these journals.[6]

### Funding

Funding for research is minimal in the low- and middleincome countries. The research agenda is low within the government health sector where competing service needs take priority.[7] Studies are donor-driven and cater to the needs of external donor agencies, thus rarely addressing national needs. A few pharmaceutical industry sponsored trials are now being conducted, and we may have to depend on such funding, until funding can be secured from sources that do not have conflict of interest. In such a backdrop, few psychiatrists have the motivation to conduct research.

### Research priorities

Most studies (80%) in the low- and middle-income countries are on the epidemiological, social, psychological, and clinical aspects.[8] Epidemiological surveys require relatively low funding. Even in this instance difficulties are encountered, due to the paucity of validated research tools and instruments in the local languages.[9] Unless internationally accepted instruments are applied the research will not be published in indexed journals. Copyright issues often preclude translation and validation of commonly used scales.

The much needed systems research, which looks at service delivery, is often abandoned due to lack of funding. Conflicting political interests prevent even the ones that are conducted from being published, if they have the potential to implicate the government in providing shoddy service.

Pharmaceutical funding is usually for drug trials. Here again the supporting industry will have profits in mind and will not necessarily include cheap but effective medications, which may not bring in profits, in the trials.[10]

The huge costs of randomized control trials when conducted in the high-income countries have no doubt made certain multinational companies look the way of the Indian subcontinent where patient recruitment into studies can be done at a fairly low cost. This, however, brings with it a range of ethical issues.

### Ethical issues

In recent times, concerns have been expressed with regard to patient coercion in research.[11] Coercion can take many forms, both overt such as financial incentives and benefits and covert, such as, clinical advantages in relation to access to services.[12] Psychiatric patients can be particularly vulnerable and the grave abuse of psychiatric patients in research is too well known to be ignored.

The need to develop strong ethics review committees, which conform to international guidelines and adhere to Good Clinical Practice guidelines on the constitution of the committees, cannot be underemphasized. A national clinical trial registry is a must in every country, in the south Asian region. The Sri Lanka Medical Association has in the past five years established this registry wherein every trial conducted in Sri Lanka has to be registered. Appropriate national legislation has to be drafted to support the conduct of clinical research within a safe ethical framework.

### Cultural aspects of research in Sri Lanka and India

The attitude of the populace to research will need to also be looked at in the context of our cultures. India and Sri Lanka have a common social value system, derived from shared religious beliefs, namely Hinduism and Buddhism. Causation of Illness is believed to be due to Karma (past action). Participating in alleviating this sorrow is seen as meritorious, leading one on the path to Nirvana (sorrowless state).

Research demands the altruistic participation of individuals, with the continuance of science in mind. Altruism is part of Buddhism, as doing good with an altruistic motive is considered as a virtue. Such beliefs may not necessarily extend to the realm of research, as scientific inquiry does not give tangible findings, the application of which the person is able to see immediately. It may take many years before any meaning is made of the process. Further, the belief in Karma, could lead to passive acceptance of disease states. Such a mind set is an impediment to research and may have to be challenged and changed.

### Partnerships

To develop research capacity, collaboration is possibly the way forward. Partnerships between the academicians and clinicians; the public and the private sectors; and the intraand inter-regional experts will have to be forged. Efforts should be made to develop consensus at the regional level with regard to the research agenda that is necessary to support health system objectives in the region. The mental health research mapping group found that the important criteria for prioritizing research were, burden of disease, social justice, and availability of funds.[8]

The formation of the SAARC Psychiatric Federation is indeed a boost to regional cooperation and collaboration. Sri Lanka has, through this network, formed many partnerships that will be mutually beneficial. The participation and contribution of academicians from India at the Annual Conferences of the Sri Lanka College of Psychiatrists over the past few years need special mention. Their insights and direction have been valuable to psychiatric trainees in Sri Lanka. It has also led to training opportunities in centers such as the National Institute of Mental Health and Neurosciences (NIMHANS), Bangalore. Many Sri Lankan psychiatrists have also had the opportunity to participate in Conferences in India.

## CONCLUSION

The dawn of the new millennium no doubt has shown a flurry of publications from the region in indexed journals, and the upward trend will hopefully continue.[13] The challenge is then to institutionalize the trend and march forward.[14]

It is also the time to lobby policymakers to not just support research in psychiatry, but also to adopt mechanisms to integrate the findings in the decision-making. Research in psychiatry is not an option. It is a dire necessity in India and Sri Lanka.
